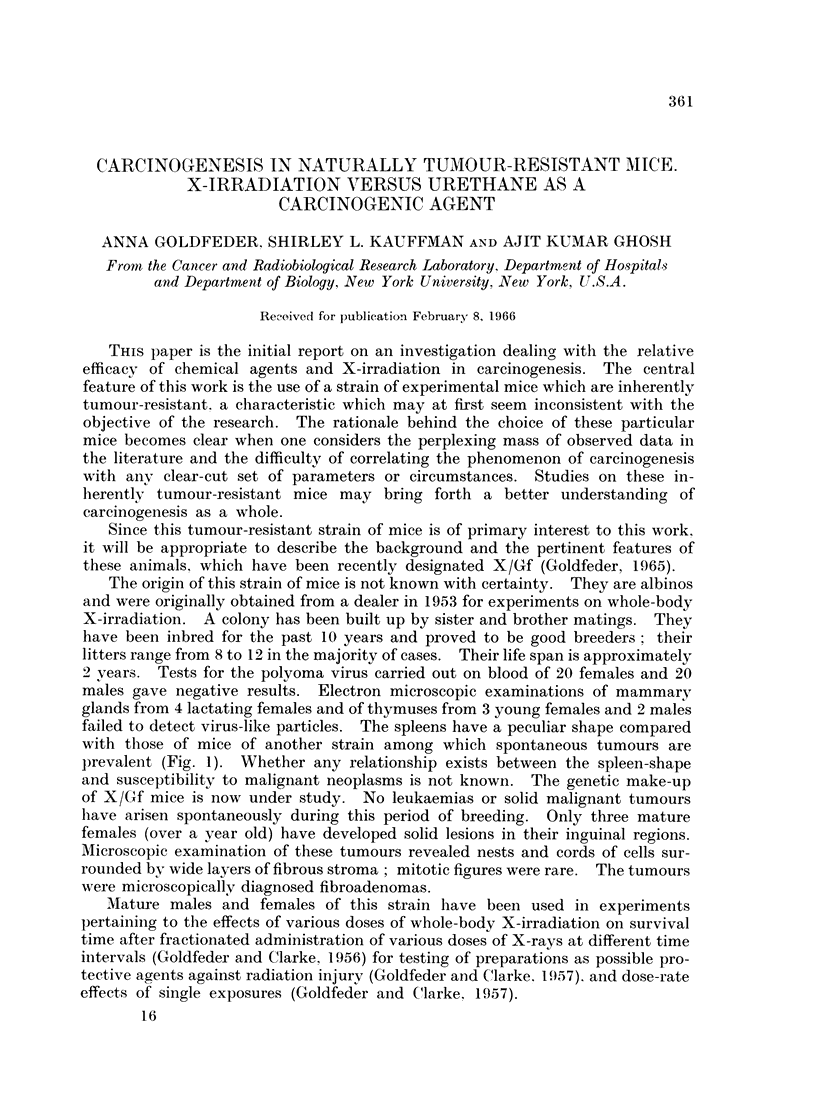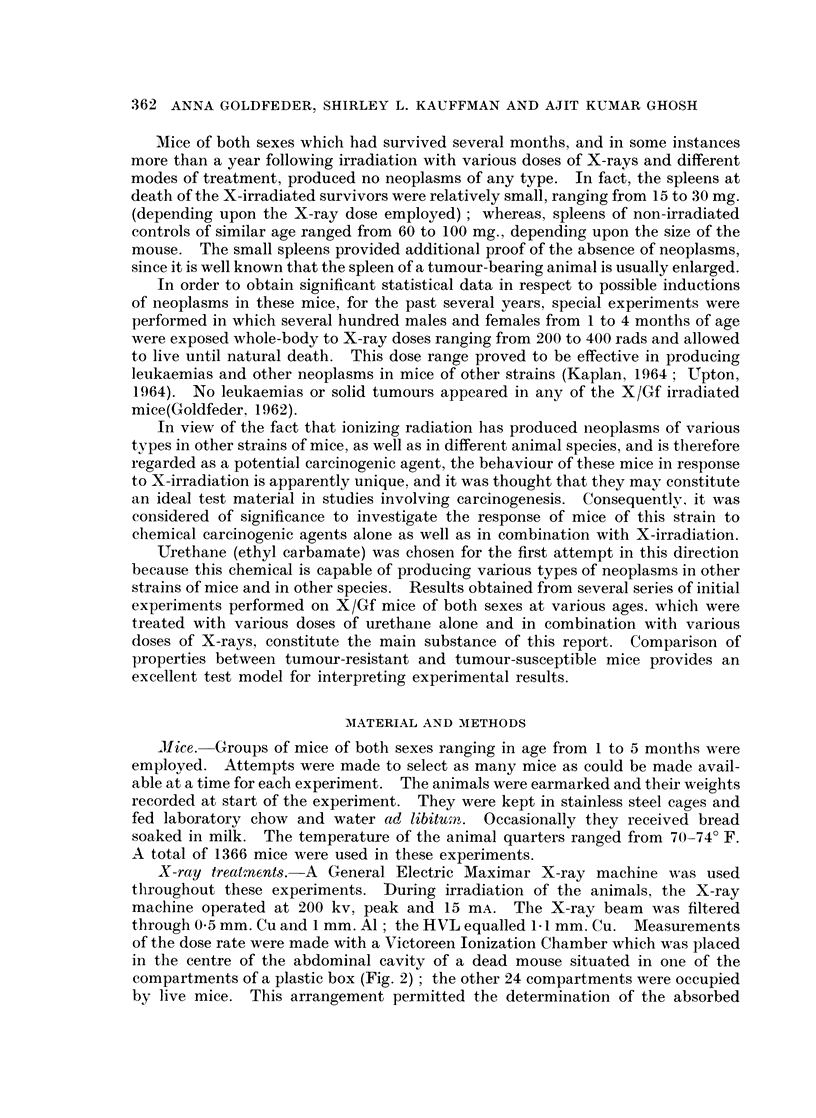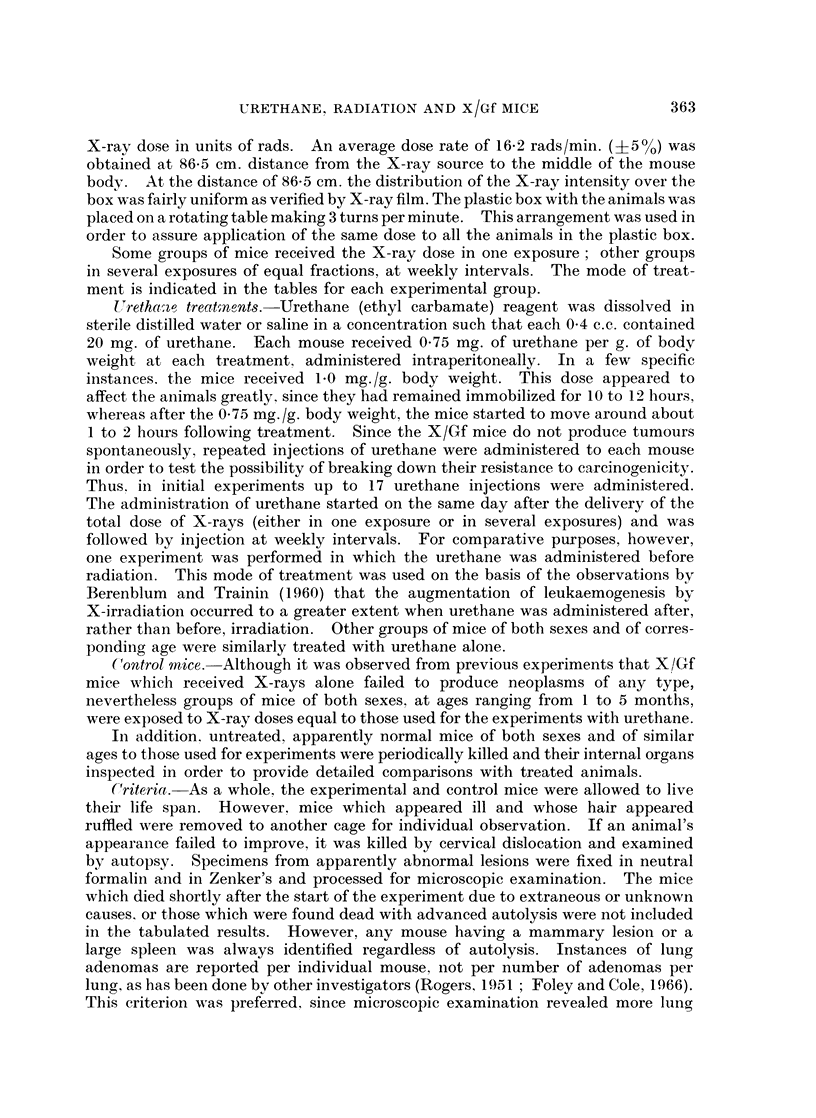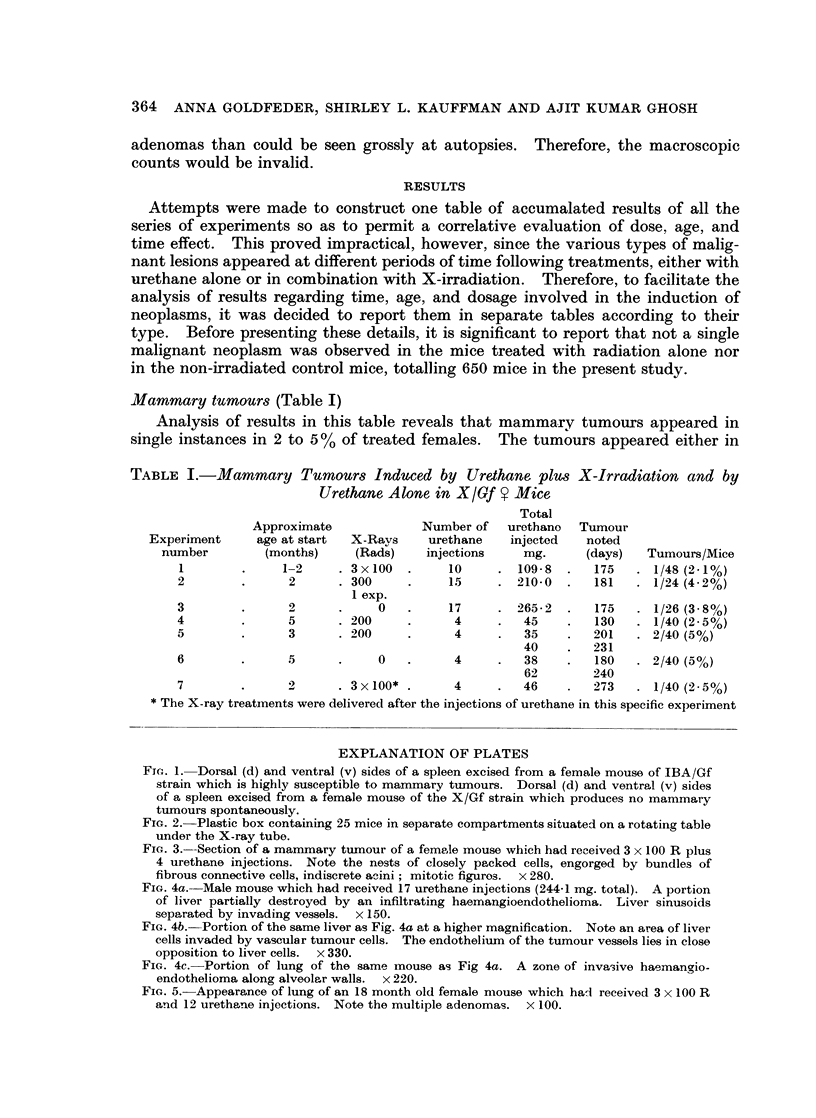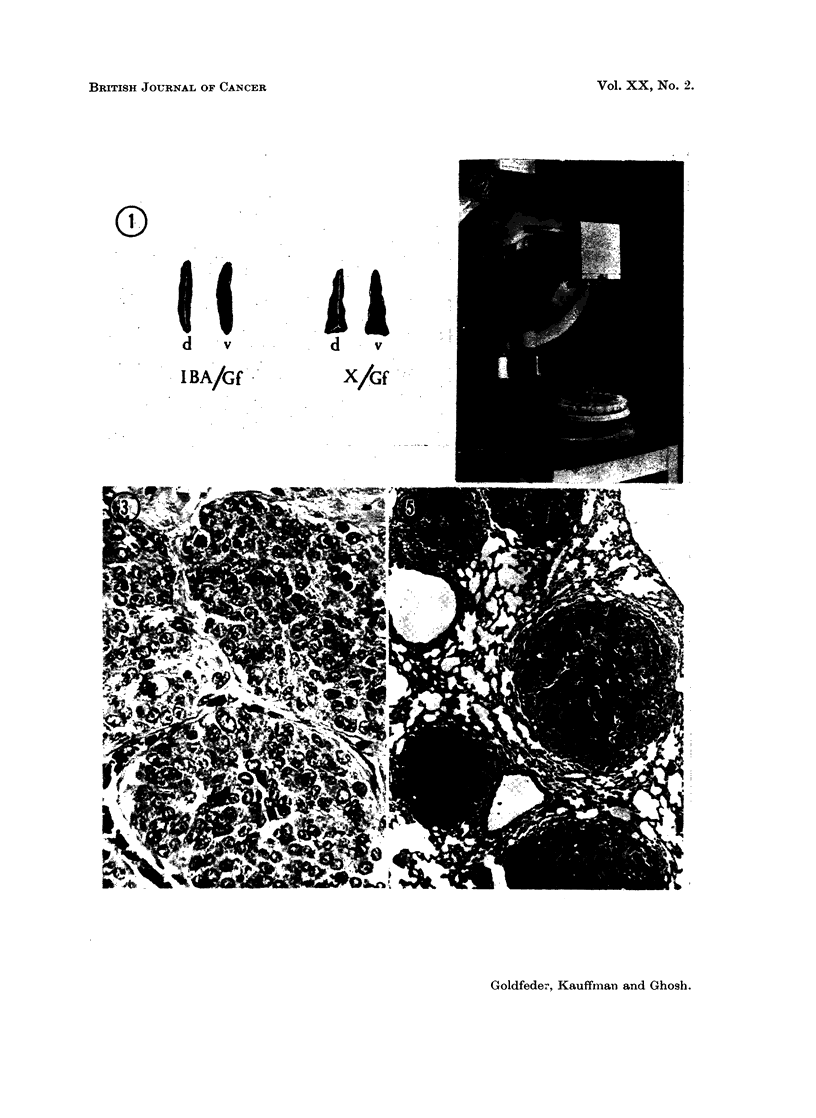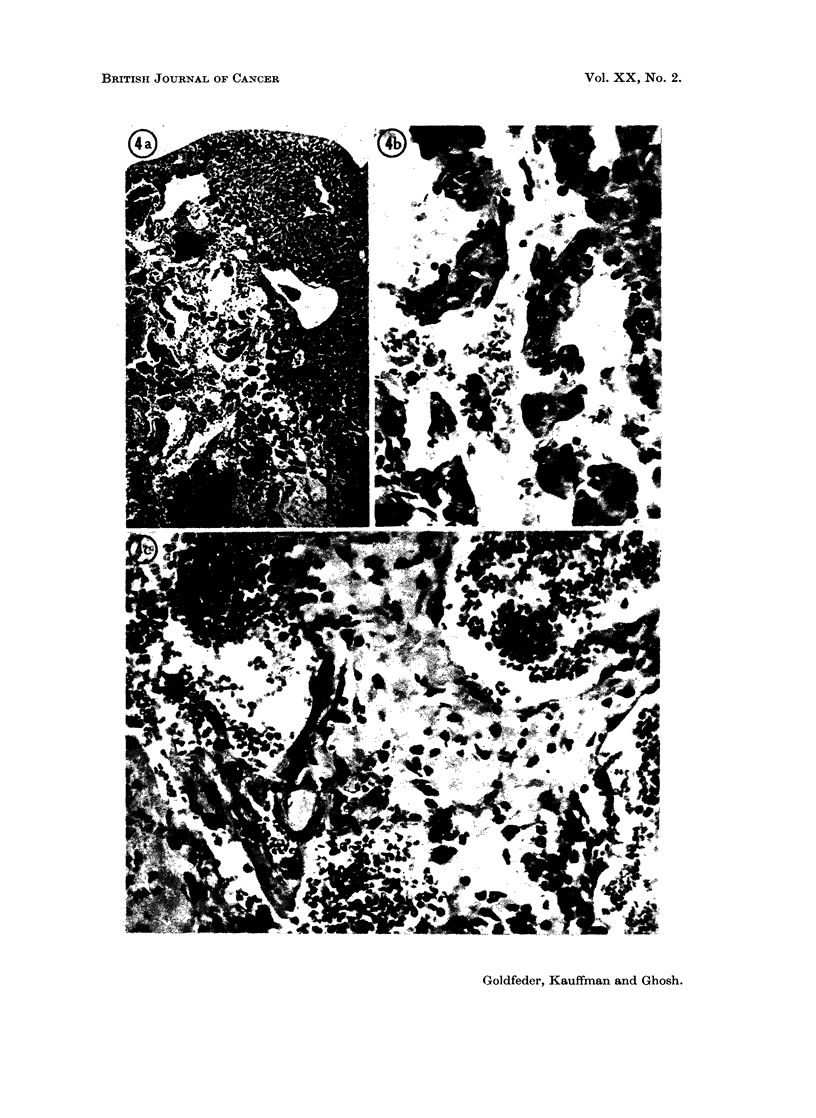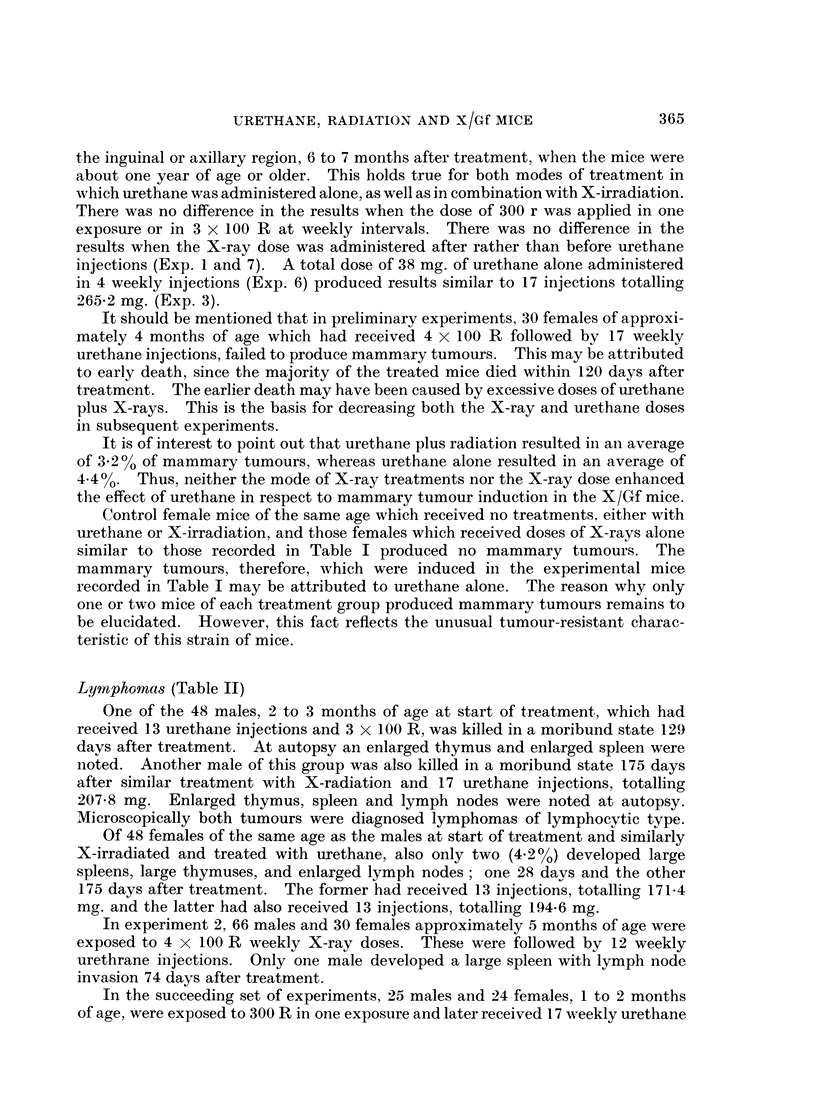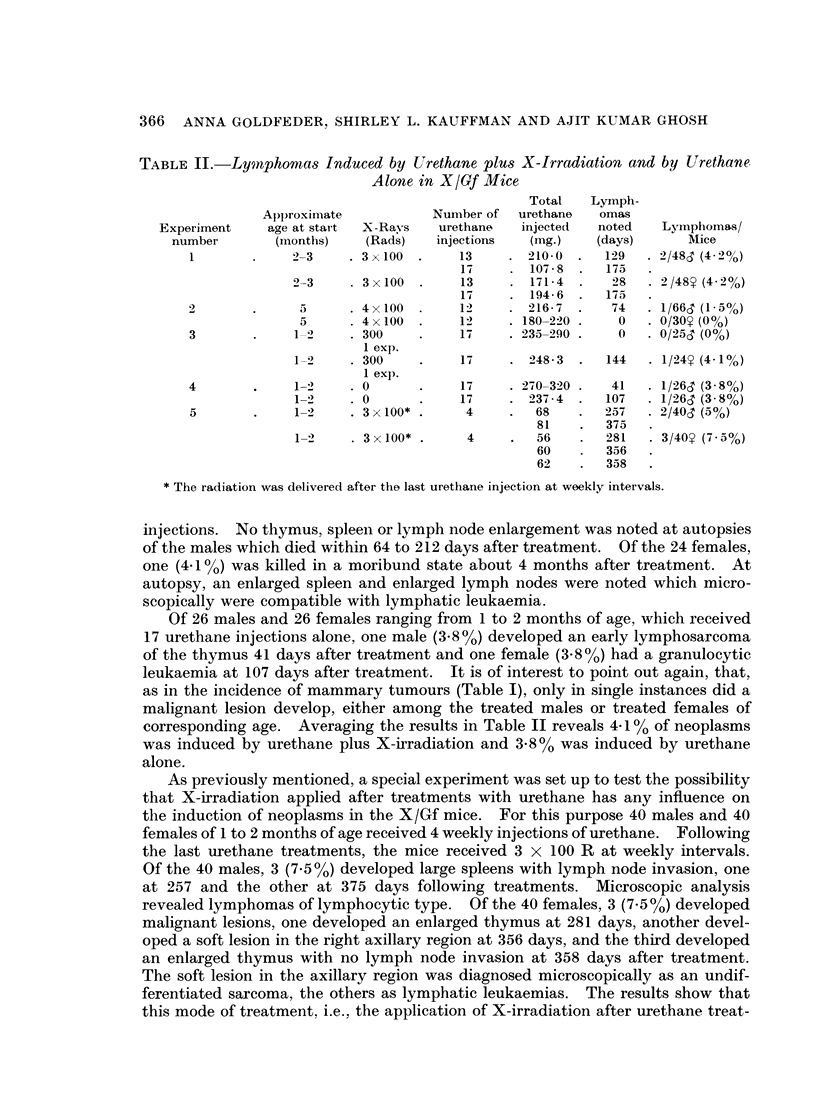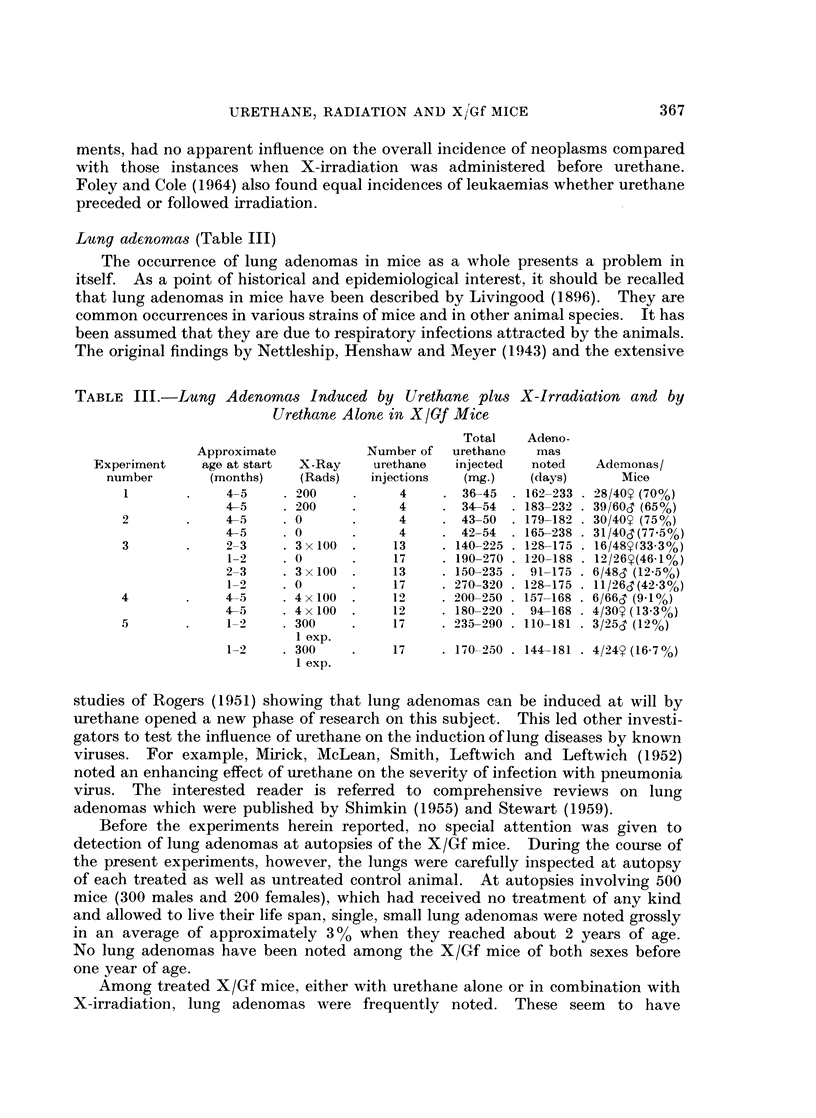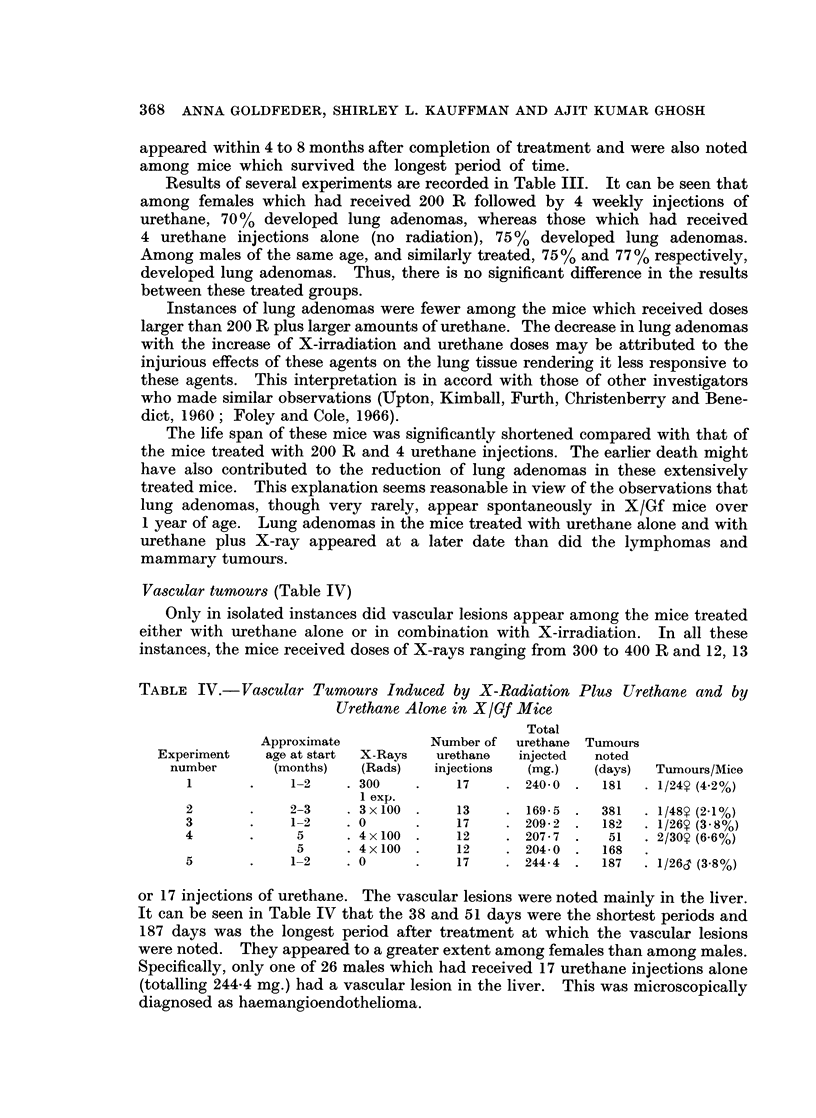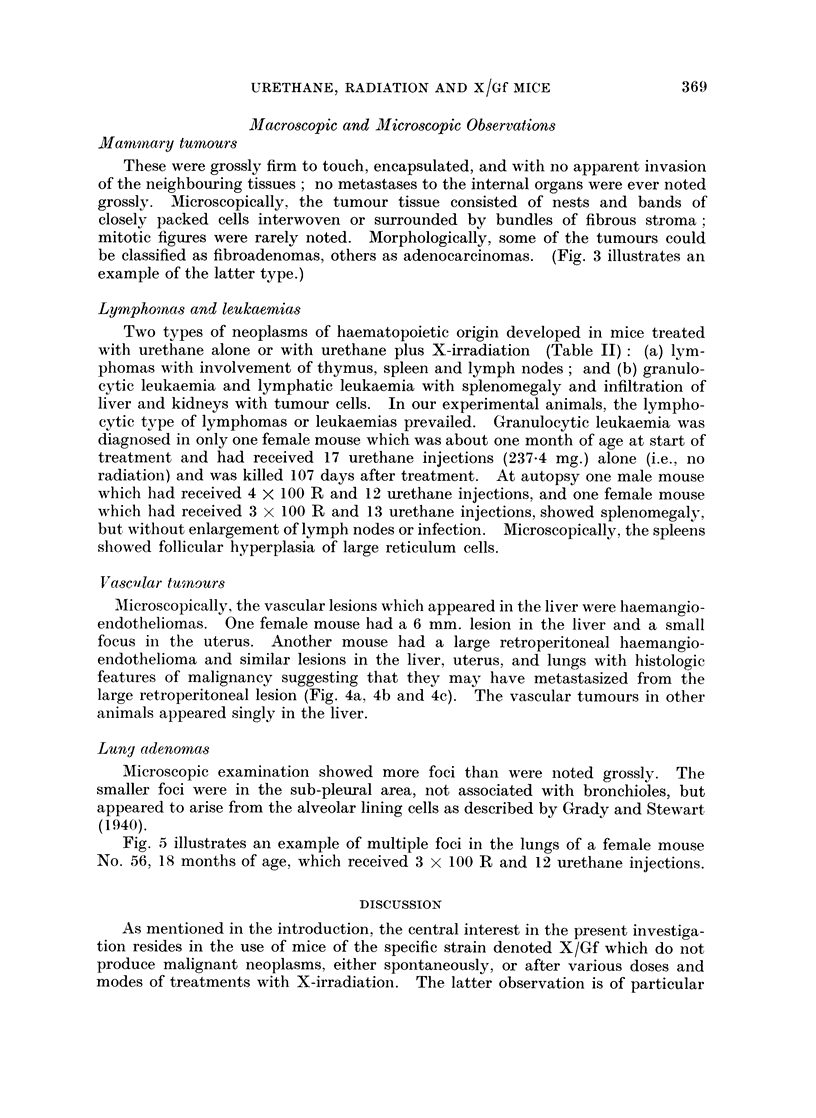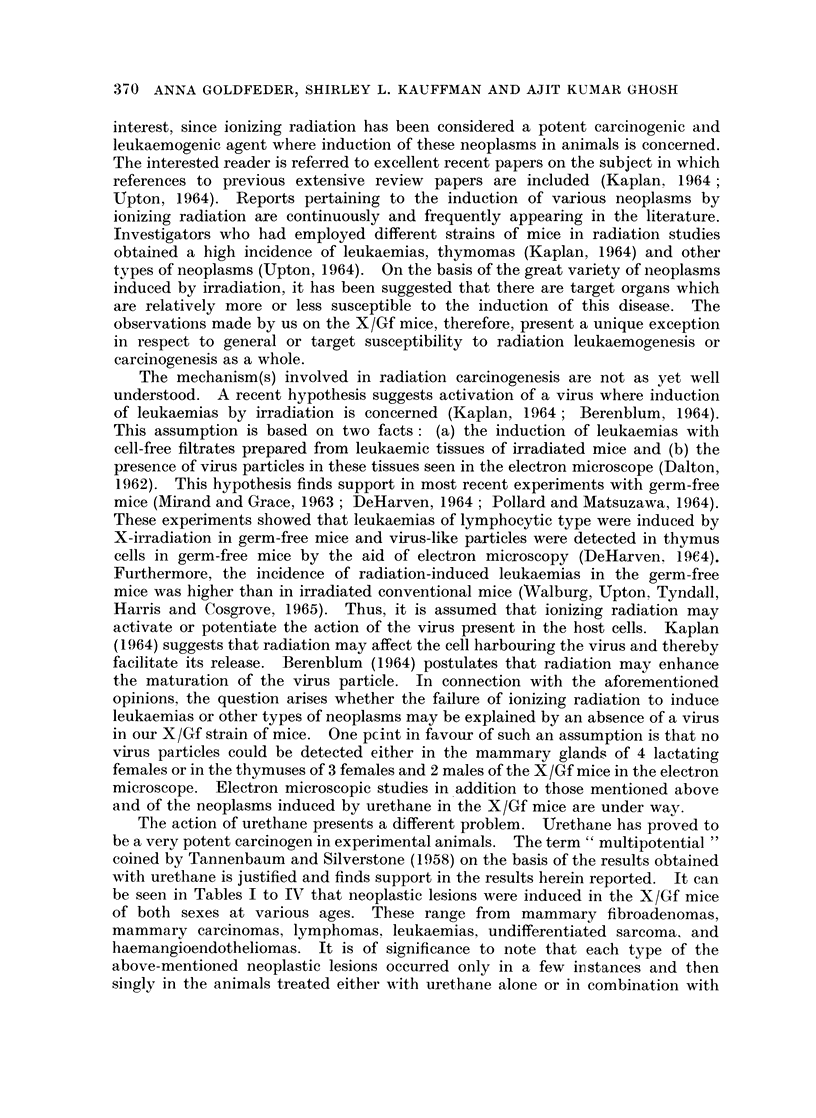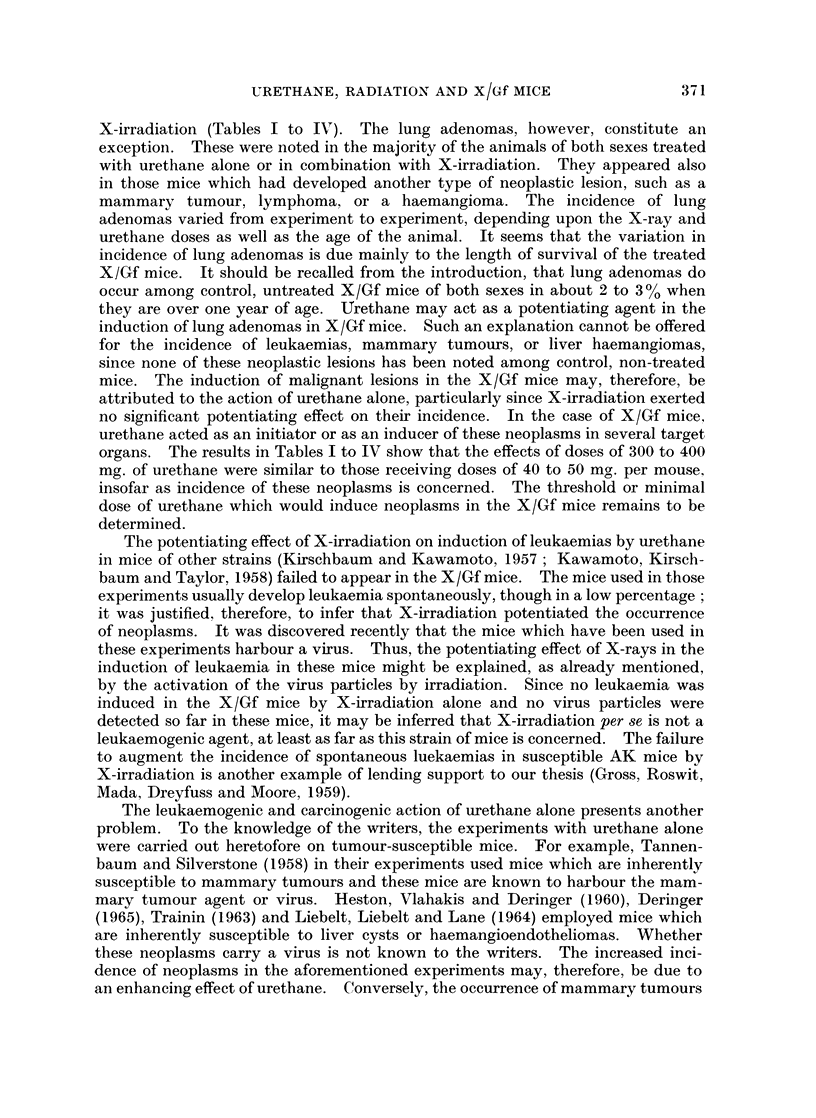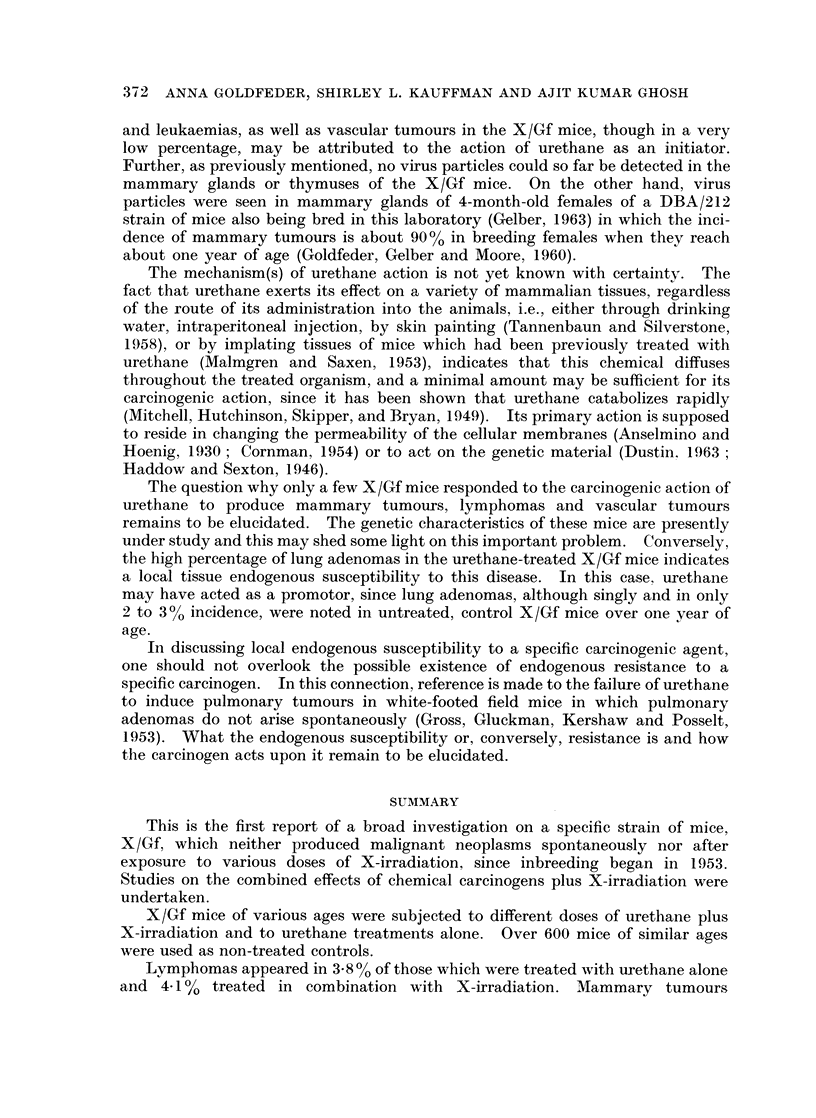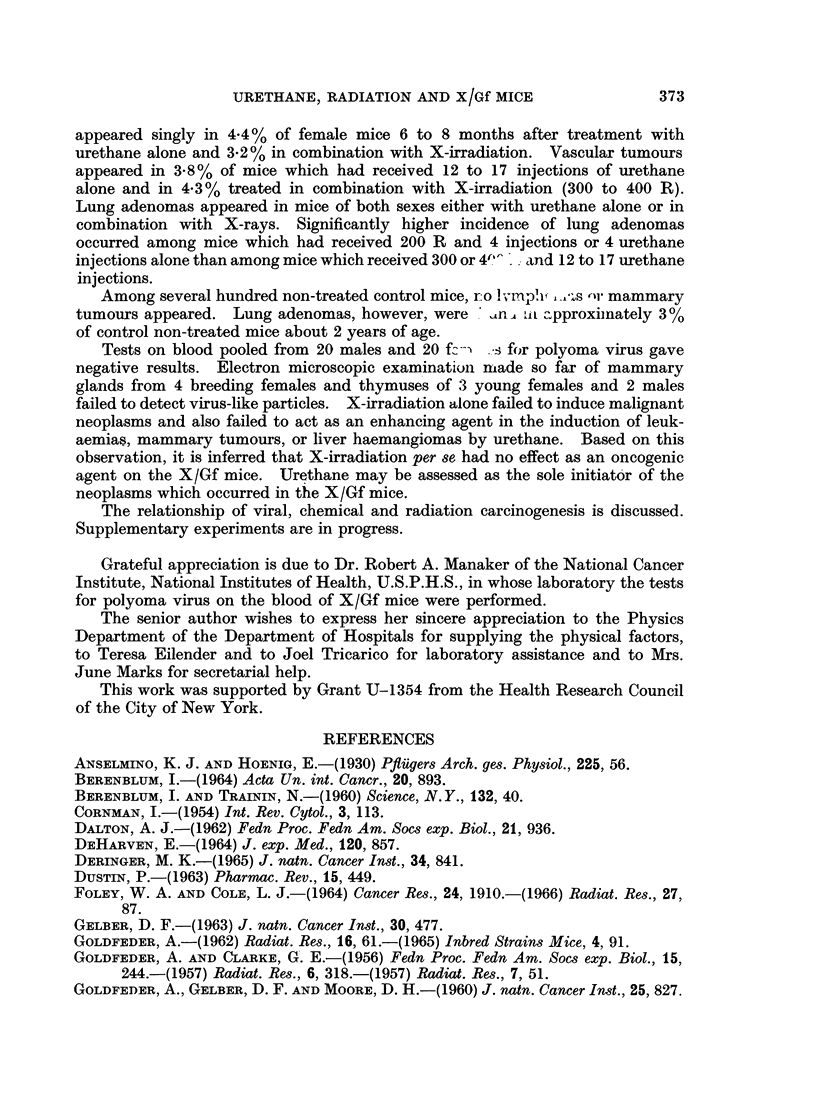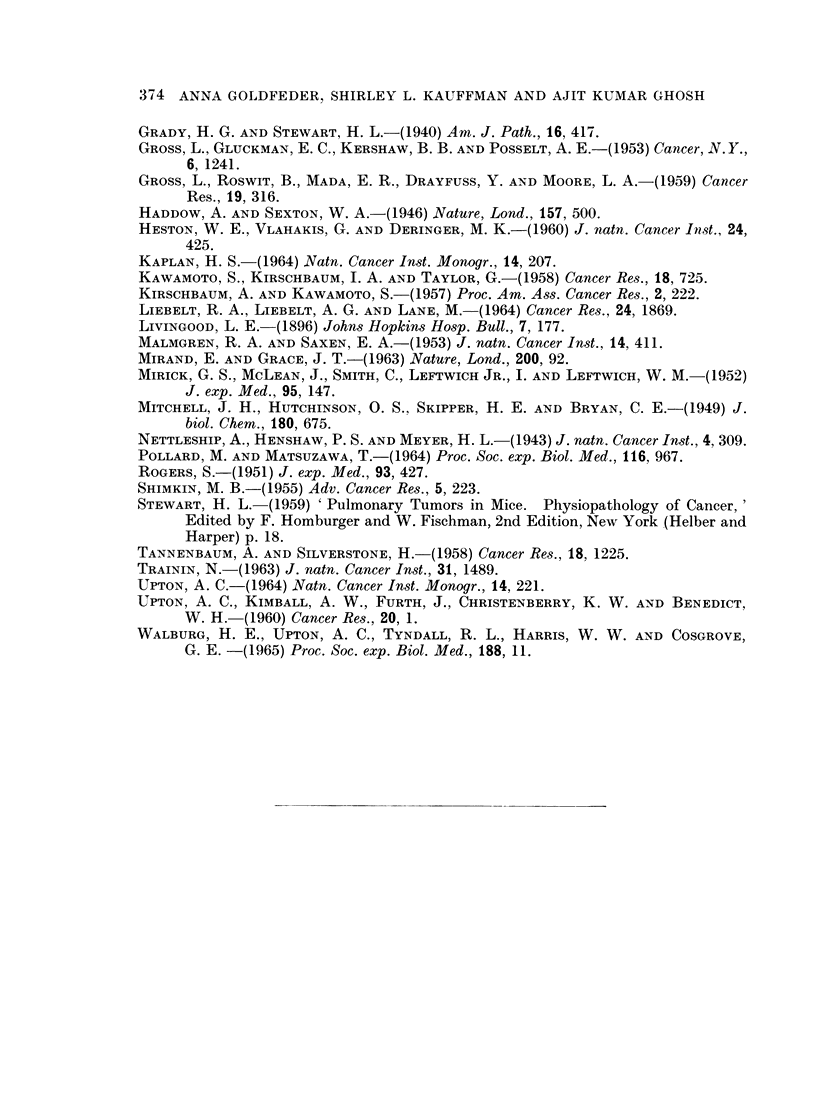# Carcinogenesis in naturally tumour-resistant mice. X-irradiation versus urethane as a carcinogenic agent.

**DOI:** 10.1038/bjc.1966.44

**Published:** 1966-06

**Authors:** A. Goldfeder, S. L. Kauffman, A. K. Ghosh

## Abstract

**Images:**


### CARCINOGENESIS IN NATURALLY TUMOUR-RESISTANT MICE. X-IRRADIATION VERSUS URETHANE AS A CARCINOGENIC AGENT

ANNA GOLDFEDER, SHIRLEY L. KAUFFMAN AND AJIT KUMAR GHOSH

*From the Cancer and Radiobiological Research Laboratory, Department of Hospitals and Department of Biology, New York University, New York, U.S.A.*

Received for publication February 8, 1966

THIS paper is the initial report on an investigation dealing with the relative efficacy of chemical agents and X-irradiation in carcinogenesis. The central feature of this work is the use of a strain of experimental mice which are inherently tumour-resistant, a characteristic which may at first seem inconsistent with the objective of the research. The rationale behind the choice of these particular mice becomes clear when one considers the perplexing mass of observed data in the literature and the difficulty of correlating the phenomenon of carcinogenesis with any clear-cut set of parameters or circumstances. Studies on these inherently tumour-resistant mice may bring forth a better understanding of carcinogenesis as a whole.

Since this tumour-resistant strain of mice is of primary interest to this work, it will be appropriate to describe the background and the pertinent features of these animals, which have been recently designated X/Gf (Goldfeder, 1965).

The origin of this strain of mice is not known with certainty. They are albinos and were originally obtained from a dealer in 1953 for experiments on whole-body X-irradiation. A colony has been built up by sister and brother matings. They have been inbred for the past 10 years and proved to be good breeders ; their litters range from 8 to 12 in the majority of cases. Their life span is approximately 2 years. Tests for the polyoma virus carried out on blood of 20 females and 20 males gave negative results. Electron microscopic examinations of mammary glands from 4 lactating females and of thymuses from 3 young females and 2 males failed to detect virus-like particles. The spleens have a peculiar shape compared with those of mice of another strain among which spontaneous tumours are prevalent (Fig. 1). Whether any relationship exists between the spleen-shape and susceptibility to malignant neoplasms is not known. The genetic make-up of X/Gf mice is now under study. No leukaemias or solid malignant tumours have arisen spontaneously during this period of breeding. Only three mature females (over a year old) have developed solid lesions in their inguinal regions. Microscopic examination of these tumours revealed nests and cords of cells surrounded by wide layers of fibrous stroma ; mitotic figures were rare. The tumours were microscopically diagnosed fibroadenomas.

Mature males and females of this strain have been used in experiments pertaining to the effects of various doses of whole-body X-irradiation on survival time after fractionated administration of various doses of X-rays at different time intervals (Goldfeder and Clarke, 1956) for testing of preparations as possible protective agents against radiation injury (Goldfeder and Clarke, 1957), and dose-rate effects of single exposures (Goldfeder and Clarke, 1957).

Mice of both sexes which had survived several months, and in some instances more than a year following irradiation with various doses of X-rays and different modes of treatment, produced no neoplasms of any type. In fact, the spleens at death of the X-irradiated survivors were relatively small, ranging from 15 to 30 mg. (depending upon the X-ray dose employed) ; whereas, spleens of non-irradiated controls of similar age ranged from 60 to 100 mg., depending upon the size of the mouse. The small spleens provided additional proof of the absence of neoplasms, since it is well known that the spleen of a tumour-bearing animal is usually enlarged.

In order to obtain significant statistical data in respect to possible inductions of neoplasms in these mice, for the past several years, special experiments were performed in which several hundred males and females from 1 to 4 months of age were exposed whole-body to X-ray doses ranging from 200 to 400 rads and allowed to live until natural death. This dose range proved to be effective in producing leukaemias and other neoplasms in mice of other strains (Kaplan, 1964 ; Upton, 1964). No leukaemias or solid tumours appeared in any of the X/Gf irradiated mice(Goldfeder, 1962).

In view of the fact that ionizing radiation has produced neoplasms of various types in other strains of mice, as well as in different animal species, and is therefore regarded as a potential carcinogenic agent, the behaviour of these mice in response to X-irradiation is apparently unique, and it was thought that they may constitute an ideal test material in studies involving carcinogenesis. Consequently, it was considered of significance to investigate the response of mice of this strain to chemical carcinogenic agents alone as well as in combination with X-irradiation.

Urethane (ethyl carbamate) was chosen for the first attempt in this direction because this chemical is capable of producing various types of neoplasms in other strains of mice and in other species. Results obtained from several series of initial experiments performed on X/Gf mice of both sexes at various ages, which were treated with various doses of urethane alone and in combination with various doses of X-rays, constitute the main substance of this report. Comparison of properties between tumour-resistant and tumour-susceptible mice provides an excellent test model for interpreting experimental results.

### MATERIAL AND METHODS

*Mice.*—Groups of mice of both sexes ranging in age from 1 to 5 months were employed. Attempts were made to select as many mice as could be made available at a time for each experiment. The animals were earmarked and their weights recorded at start of the experiment. They were kept in stainless steel cages and fed laboratory chow and water *ad libitum*. Occasionally they received bread soaked in milk. The temperature of the animal quarters ranged from 70–74° F. A total of 1366 mice were used in these experiments.

*X-ray treatments.*—A General Electric Maximar X-ray machine was used throughout these experiments. During irradiation of the animals, the X-ray machine operated at 200 kv, peak and 15 mA. The X-ray beam was filtered through 0·5 mm. Cu and 1 mm. Al ; the HVL equalled 1·1 mm. Cu. Measurements of the dose rate were made with a Victoreen Ionization Chamber which was placed in the centre of the abdominal cavity of a dead mouse situated in one of the compartments of a plastic box (Fig. 2) ; the other 24 compartments were occupied by live mice. This arrangement permitted the determination of the absorbed

X-ray dose in units of rads. An average dose rate of 16·2 rads/min. ($\pm 5\%$) was obtained at 86·5 cm. distance from the X-ray source to the middle of the mouse body. At the distance of 86·5 cm. the distribution of the X-ray intensity over the box was fairly uniform as verified by X-ray film. The plastic box with the animals was placed on a rotating table making 3 turns per minute. This arrangement was used in order to assure application of the same dose to all the animals in the plastic box.

Some groups of mice received the X-ray dose in one exposure ; other groups in several exposures of equal fractions, at weekly intervals. The mode of treatment is indicated in the tables for each experimental group.

*Urethane treatments.*—Urethane (ethyl carbamate) reagent was dissolved in sterile distilled water or saline in a concentration such that each 0·4 c.c. contained 20 mg. of urethane. Each mouse received 0·75 mg. of urethane per g. of body weight at each treatment, administered intraperitoneally. In a few specific instances, the mice received 1·0 mg./g. body weight. This dose appeared to affect the animals greatly, since they had remained immobilized for 10 to 12 hours, whereas after the 0·75 mg./g. body weight, the mice started to move around about 1 to 2 hours following treatment. Since the X/Gf mice do not produce tumours spontaneously, repeated injections of urethane were administered to each mouse in order to test the possibility of breaking down their resistance to carcinogenicity. Thus, in initial experiments up to 17 urethane injections were administered. The administration of urethane started on the same day after the delivery of the total dose of X-rays (either in one exposure or in several exposures) and was followed by injection at weekly intervals. For comparative purposes, however, one experiment was performed in which the urethane was administered before radiation. This mode of treatment was used on the basis of the observations by Berenblum and Trainin (1960) that the augmentation of leukaemogenesis by X-irradiation occurred to a greater extent when urethane was administered after, rather than before, irradiation. Other groups of mice of both sexes and of corresponding age were similarly treated with urethane alone.

*Control mice.*—Although it was observed from previous experiments that X/Gf mice which received X-rays alone failed to produce neoplasms of any type, nevertheless groups of mice of both sexes, at ages ranging from 1 to 5 months, were exposed to X-ray doses equal to those used for the experiments with urethane.

In addition, untreated, apparently normal mice of both sexes and of similar ages to those used for experiments were periodically killed and their internal organs inspected in order to provide detailed comparisons with treated animals.

*Criteria.*—As a whole, the experimental and control mice were allowed to live their life span. However, mice which appeared ill and whose hair appeared ruffled were removed to another cage for individual observation. If an animal's appearance failed to improve, it was killed by cervical dislocation and examined by autopsy. Specimens from apparently abnormal lesions were fixed in neutral formalin and in Zenker's and processed for microscopic examination. The mice which died shortly after the start of the experiment due to extraneous or unknown causes, or those which were found dead with advanced autolysis were not included in the tabulated results. However, any mouse having a mammary lesion or a large spleen was always identified regardless of autolysis. Instances of lung adenomas are reported per individual mouse, not per number of adenomas per lung, as has been done by other investigators (Rogers, 1951 ; Foley and Cole, 1966). This criterion was preferred, since microscopic examination revealed more lung adenomas than could be seen grossly at autopsies. Therefore, the macroscopic counts would be invalid.

### RESULTS

Attempts were made to construct one table of accumalated results of all the series of experiments so as to permit a correlative evaluation of dose, age, and time effect. This proved impractical, however, since the various types of malignant lesions appeared at different periods of time following treatments, either with urethane alone or in combination with X-irradiation. Therefore, to facilitate the analysis of results regarding time, age, and dosage involved in the induction of neoplasms, it was decided to report them in separate tables according to their type. Before presenting these details, it is significant to report that not a single malignant neoplasm was observed in the mice treated with radiation alone nor in the non-irradiated control mice, totalling 650 mice in the present study.

*Mammary tumours* (Table I)

Analysis of results in this table reveals that mammary tumours appeared in single instances in 2 to 5% of treated females. The tumours appeared either in

TABLE I.—*Mammary Tumours Induced by Urethane plus X-Irradiation and by Urethane Alone in X/Gf ♀ Mice*

| Experiment number | Approximate age at start (months) | X-Rays (Rads) | Number of urethane injections | Total urethane injected mg. | Tumour noted (days) | Tumours/Mice |
|---|---|---|---|---|---|---|
| 1 | 1–2 | 3×100 | 10 | 109·8 | 175 | 1/48 (2·1%) |
| 2 | 2 | 300<br>1 exp. | 15 | 210·0 | 181 | 1/24 (4·2%) |
| 3 | 2 | 0 | 17 | 265·2 | 175 | 1/26 (3·8%) |
| 4 | 5 | 200 | 4 | 45 | 130 | 1/40 (2·5%) |
| 5 | 3 | 200 | 4 | 35<br>40 | 201<br>231 | 2/40 (5%) |
| 6 | 5 | 0 | 4 | 38<br>62 | 180<br>240 | 2/40 (5%) |
| 7 | 2 | 3×100* | 4 | 46 | 273 | 1/40 (2·5%) |

* The X-ray treatments were delivered after the injections of urethane in this specific experiment

### EXPLANATION OF PLATES

FIG. 1.—Dorsal (d) and ventral (v) sides of a spleen excised from a female mouse of IBA/Gf strain which is highly susceptible to mammary tumours. Dorsal (d) and ventral (v) sides of a spleen excised from a female mouse of the X/Gf strain which produces no mammary tumours spontaneously.

FIG. 2.—Plastic box containing 25 mice in separate compartments situated on a rotating table under the X-ray tube.

FIG. 3.—Section of a mammary tumour of a female mouse which had received 3 × 100 R plus 4 urethane injections. Note the nests of closely packed cells, engorged by bundles of fibrous connective cells, indiscrete acini ; mitotic figuros. × 280.

FIG. 4*a*.—Male mouse which had received 17 urethane injections (244·1 mg. total). A portion of liver partially destroyed by an infiltrating haemangioendothelioma. Liver sinusoids separated by invading vessels. × 150.

FIG. 4*b*.—Portion of the same liver as Fig. 4*a* at a higher magnification. Note an area of liver cells invaded by vascular tumour cells. The endothelium of the tumour vessels lies in close opposition to liver cells. × 330.

FIG. 4*c*.—Portion of lung of the same mouse as Fig 4*a*. A zone of invasive haemangio-endothelioma along alveolar walls. × 220.

FIG. 5.—Appearance of lung of an 18 month old female mouse which had received 3 × 100 R and 12 urethane injections. Note the multiple adenomas. × 100.

Goldfeder, Kauffman and Ghosh.

Goldfeder, Kauffman and Ghosh.

the inguinal or axillary region, 6 to 7 months after treatment, when the mice were about one year of age or older. This holds true for both modes of treatment in which urethane was administered alone, as well as in combination with X-irradiation. There was no difference in the results when the dose of 300 r was applied in one exposure or in 3 × 100 R at weekly intervals. There was no difference in the results when the X-ray dose was administered after rather than before urethane injections (Exp. 1 and 7). A total dose of 38 mg. of urethane alone administered in 4 weekly injections (Exp. 6) produced results similar to 17 injections totalling 265·2 mg. (Exp. 3).

It should be mentioned that in preliminary experiments, 30 females of approximately 4 months of age which had received 4 × 100 R followed by 17 weekly urethane injections, failed to produce mammary tumours. This may be attributed to early death, since the majority of the treated mice died within 120 days after treatment. The earlier death may have been caused by excessive doses of urethane plus X-rays. This is the basis for decreasing both the X-ray and urethane doses in subsequent experiments.

It is of interest to point out that urethane plus radiation resulted in an average of 3·2% of mammary tumours, whereas urethane alone resulted in an average of 4·4%. Thus, neither the mode of X-ray treatments nor the X-ray dose enhanced the effect of urethane in respect to mammary tumour induction in the X/Gf mice.

Control female mice of the same age which received no treatments, either with urethane or X-irradiation, and those females which received doses of X-rays alone similar to those recorded in Table I produced no mammary tumours. The mammary tumours, therefore, which were induced in the experimental mice recorded in Table I may be attributed to urethane alone. The reason why only one or two mice of each treatment group produced mammary tumours remains to be elucidated. However, this fact reflects the unusual tumour-resistant characteristic of this strain of mice.

*Lymphomas* (Table II)

One of the 48 males, 2 to 3 months of age at start of treatment, which had received 13 urethane injections and 3 × 100 R, was killed in a moribund state 129 days after treatment. At autopsy an enlarged thymus and enlarged spleen were noted. Another male of this group was also killed in a moribund state 175 days after similar treatment with X-radiation and 17 urethane injections, totalling 207·8 mg. Enlarged thymus, spleen and lymph nodes were noted at autopsy. Microscopically both tumours were diagnosed lymphomas of lymphocytic type.

Of 48 females of the same age as the males at start of treatment and similarly X-irradiated and treated with urethane, also only two (4·2%) developed large spleens, large thymuses, and enlarged lymph nodes ; one 28 days and the other 175 days after treatment. The former had received 13 injections, totalling 171·4 mg. and the latter had also received 13 injections, totalling 194·6 mg.

In experiment 2, 66 males and 30 females approximately 5 months of age were exposed to 4 × 100 R weekly X-ray doses. These were followed by 12 weekly urethrane injections. Only one male developed a large spleen with lymph node invasion 74 days after treatment.

In the succeeding set of experiments, 25 males and 24 females, 1 to 2 months of age, were exposed to 300 R in one exposure and later received 17 weekly urethane

TABLE II.—*Lymphomas Induced by Urethane plus X-Irradiation and by Urethane Alone in X/Gf Mice*

| Experiment number | Approximate age at start (months) | X-Rays (Rads) | Number of urethane injections | Total urethane injected (mg.) | Lymphomas noted (days) | Lymphomas/Mice |
|---|---|---|---|---|---|---|
| 1 | 2–3 | 3 × 100 | 13 | 210·0 | 129 | 2/48♂ (4·2%) |
| | | | 17 | 107·8 | 175 | |
| | 2–3 | 3 × 100 | 13 | 171·4 | 28 | 2/48♀ (4·2%) |
| | | | 17 | 194·6 | 175 | |
| 2 | 5 | 4 × 100 | 12 | 216·7 | 74 | 1/66♂ (1·5%) |
| | 5 | 4 × 100 | 12 | 180–220 | 0 | 0/30♀ (0%) |
| 3 | 1–2 | 300 1 exp. | 17 | 235–290 | 0 | 0/25♂ (0%) |
| | 1–2 | 300 1 exp. | 17 | 248·3 | 144 | 1/24♀ (4·1%) |
| 4 | 1–2 | 0 | 17 | 270–320 | 41 | 1/26♂ (3·8%) |
| | 1–2 | 0 | 17 | 237·4 | 107 | 1/26♂ (3·8%) |
| 5 | 1–2 | 3 × 100* | 4 | 68 | 257 | 2/40♂ (5%) |
| | | | | 81 | 375 | |
| | 1–2 | 3 × 100* | 4 | 56 | 281 | 3/40♀ (7·5%) |
| | | | | 60 | 356 | |
| | | | | 62 | 358 | |

* The radiation was delivered after the last urethane injection at weekly intervals.

injections. No thymus, spleen or lymph node enlargement was noted at autopsies of the males which died within 64 to 212 days after treatment. Of the 24 females, one (4·1%) was killed in a moribund state about 4 months after treatment. At autopsy, an enlarged spleen and enlarged lymph nodes were noted which microscopically were compatible with lymphatic leukaemia.

Of 26 males and 26 females ranging from 1 to 2 months of age, which received 17 urethane injections alone, one male (3·8%) developed an early lymphosarcoma of the thymus 41 days after treatment and one female (3·8%) had a granulocytic leukaemia at 107 days after treatment. It is of interest to point out again, that, as in the incidence of mammary tumours (Table I), only in single instances did a malignant lesion develop, either among the treated males or treated females of corresponding age. Averaging the results in Table II reveals 4·1% of neoplasms was induced by urethane plus X-irradiation and 3·8% was induced by urethane alone.

As previously mentioned, a special experiment was set up to test the possibility that X-irradiation applied after treatments with urethane has any influence on the induction of neoplasms in the X/Gf mice. For this purpose 40 males and 40 females of 1 to 2 months of age received 4 weekly injections of urethane. Following the last urethane treatments, the mice received 3 × 100 R at weekly intervals. Of the 40 males, 3 (7·5%) developed large spleens with lymph node invasion, one at 257 and the other at 375 days following treatments. Microscopic analysis revealed lymphomas of lymphocytic type. Of the 40 females, 3 (7·5%) developed malignant lesions, one developed an enlarged thymus at 281 days, another developed a soft lesion in the right axillary region at 356 days, and the third developed an enlarged thymus with no lymph node invasion at 358 days after treatment. The soft lesion in the axillary region was diagnosed microscopically as an undifferentiated sarcoma, the others as lymphatic leukaemias. The results show that this mode of treatment, i.e., the application of X-irradiation after urethane treatments, had no apparent influence on the overall incidence of neoplasms compared with those instances when X-irradiation was administered before urethane. Foley and Cole (1964) also found equal incidences of leukaemias whether urethane preceded or followed irradiation.

*Lung adenomas* (Table III)

The occurrence of lung adenomas in mice as a whole presents a problem in itself. As a point of historical and epidemiological interest, it should be recalled that lung adenomas in mice have been described by Livingood (1896). They are common occurrences in various strains of mice and in other animal species. It has been assumed that they are due to respiratory infections attracted by the animals. The original findings by Nettleship, Henshaw and Meyer (1943) and the extensive

TABLE III.—*Lung Adenomas Induced by Urethane plus X-Irradiation and by Urethane Alone in X/Gf Mice*

| Experiment number | Approximate age at start (months) | X-Ray (Rads) | Number of urethane injections | Total urethane injected (mg.) | Adenomas noted (days) | Ademonas/ Mice |
|---|---|---|---|---|---|---|
| 1 | 4–5 | 200 | 4 | 36–45 | 162–233 | 28/40♀ (70%) |
| | 4–5 | 200 | 4 | 34–54 | 183–232 | 39/60♂ (65%) |
| 2 | 4–5 | 0 | 4 | 43–50 | 179–182 | 30/40♀ (75%) |
| | 4–5 | 0 | 4 | 42–54 | 165–238 | 31/40♂ (77·5%) |
| 3 | 2–3 | 3 × 100 | 13 | 140–225 | 128–175 | 16/48♀ (33·3%) |
| | 1–2 | 0 | 17 | 190–270 | 120–188 | 12/26♀ (46·1%) |
| | 2–3 | 3 × 100 | 13 | 150–235 | 91–175 | 6/48♂ (12·5%) |
| | 1–2 | 0 | 17 | 270–320 | 128–175 | 11/26♂ (42·3%) |
| 4 | 4–5 | 4 × 100 | 12 | 200–250 | 157–168 | 6/66♂ (9·1%) |
| | 4–5 | 4 × 100 | 12 | 180–220 | 94–168 | 4/30♀ (13·3%) |
| 5 | 1–2 | 300 1 exp. | 17 | 235–290 | 110–181 | 3/25♂ (12%) |
| | 1–2 | 300 1 exp. | 17 | 170–250 | 144–181 | 4/24♀ (16·7%) |

studies of Rogers (1951) showing that lung adenomas can be induced at will by urethane opened a new phase of research on this subject. This led other investigators to test the influence of urethane on the induction of lung diseases by known viruses. For example, Mirick, McLean, Smith, Leftwich and Leftwich (1952) noted an enhancing effect of urethane on the severity of infection with pneumonia virus. The interested reader is referred to comprehensive reviews on lung adenomas which were published by Shimkin (1955) and Stewart (1959).

Before the experiments herein reported, no special attention was given to detection of lung adenomas at autopsies of the X/Gf mice. During the course of the present experiments, however, the lungs were carefully inspected at autopsy of each treated as well as untreated control animal. At autopsies involving 500 mice (300 males and 200 females), which had received no treatment of any kind and allowed to live their life span, single, small lung adenomas were noted grossly in an average of approximately 3% when they reached about 2 years of age. No lung adenomas have been noted among the X/Gf mice of both sexes before one year of age.

Among treated X/Gf mice, either with urethane alone or in combination with X-irradiation, lung adenomas were frequently noted. These seem to have appeared within 4 to 8 months after completion of treatment and were also noted among mice which survived the longest period of time.

Results of several experiments are recorded in Table III. It can be seen that among females which had received 200 R followed by 4 weekly injections of urethane, 70% developed lung adenomas, whereas those which had received 4 urethane injections alone (no radiation), 75% developed lung adenomas. Among males of the same age, and similarly treated, 75% and 77% respectively, developed lung adenomas. Thus, there is no significant difference in the results between these treated groups.

Instances of lung adenomas were fewer among the mice which received doses larger than 200 R plus larger amounts of urethane. The decrease in lung adenomas with the increase of X-irradiation and urethane doses may be attributed to the injurious effects of these agents on the lung tissue rendering it less responsive to these agents. This interpretation is in accord with those of other investigators who made similar observations (Upton, Kimball, Furth, Christenberry and Benedict, 1960 ; Foley and Cole, 1966).

The life span of these mice was significantly shortened compared with that of the mice treated with 200 R and 4 urethane injections. The earlier death might have also contributed to the reduction of lung adenomas in these extensively treated mice. This explanation seems reasonable in view of the observations that lung adenomas, though very rarely, appear spontaneously in X/Gf mice over 1 year of age. Lung adenomas in the mice treated with urethane alone and with urethane plus X-ray appeared at a later date than did the lymphomas and mammary tumours.

*Vascular tumours* (Table IV)

Only in isolated instances did vascular lesions appear among the mice treated either with urethane alone or in combination with X-irradiation. In all these instances, the mice received doses of X-rays ranging from 300 to 400 R and 12, 13

TABLE IV.—*Vascular Tumours Induced by X-Radiation Plus Urethane and by Urethane Alone in X/Gf Mice*

| Experiment number | Approximate age at start (months) | X-Rays (Rads) | Number of urethane injections | Total urethane injected (mg.) | Tumours noted (days) | Tumours/Mice |
|---|---|---|---|---|---|---|
| 1 | 1–2 | 300 1 exp. | 17 | 240·0 | 181 | 1/24♀ (4·2%) |
| 2 | 2–3 | 3×100 | 13 | 169·5 | 381 | 1/48♀ (2·1%) |
| 3 | 1–2 | 0 | 17 | 209·2 | 182 | 1/26♀ (3·8%) |
| 4 | 5 | 4×100 | 12 | 207·7 | 51 | 2/30♀ (6·6%) |
| | 5 | 4×100 | 12 | 204·0 | 168 | |
| 5 | 1–2 | 0 | 17 | 244·4 | 187 | 1/26♂ (3·8%) |

or 17 injections of urethane. The vascular lesions were noted mainly in the liver. It can be seen in Table IV that the 38 and 51 days were the shortest periods and 187 days was the longest period after treatment at which the vascular lesions were noted. They appeared to a greater extent among females than among males. Specifically, only one of 26 males which had received 17 urethane injections alone (totalling 244·4 mg.) had a vascular lesion in the liver. This was microscopically diagnosed as haemangioendothelioma.

### *Macroscopic and Microscopic Observations*

*Mammary tumours*

These were grossly firm to touch, encapsulated, and with no apparent invasion of the neighbouring tissues ; no metastases to the internal organs were ever noted grossly. Microscopically, the tumour tissue consisted of nests and bands of closely packed cells interwoven or surrounded by bundles of fibrous stroma ; mitotic figures were rarely noted. Morphologically, some of the tumours could be classified as fibroadenomas, others as adenocarcinomas. (Fig. 3 illustrates an example of the latter type.)

*Lymphomas and leukaemias*

Two types of neoplasms of haematopoietic origin developed in mice treated with urethane alone or with urethane plus X-irradiation (Table II) : (a) lymphomas with involvement of thymus, spleen and lymph nodes ; and (b) granulocytic leukaemia and lymphatic leukaemia with splenomegaly and infiltration of liver and kidneys with tumour cells. In our experimental animals, the lymphocytic type of lymphomas or leukaemias prevailed. Granulocytic leukaemia was diagnosed in only one female mouse which was about one month of age at start of treatment and had received 17 urethane injections (237·4 mg.) alone (i.e., no radiation) and was killed 107 days after treatment. At autopsy one male mouse which had received 4 × 100 R and 12 urethane injections, and one female mouse which had received 3 × 100 R and 13 urethane injections, showed splenomegaly, but without enlargement of lymph nodes or infection. Microscopically, the spleens showed follicular hyperplasia of large reticulum cells.

*Vascular tumours*

Microscopically, the vascular lesions which appeared in the liver were haemangioendotheliomas. One female mouse had a 6 mm. lesion in the liver and a small focus in the uterus. Another mouse had a large retroperitoneal haemangioendothelioma and similar lesions in the liver, uterus, and lungs with histologic features of malignancy suggesting that they may have metastasized from the large retroperitoneal lesion (Fig. 4a, 4b and 4c). The vascular tumours in other animals appeared singly in the liver.

*Lung adenomas*

Microscopic examination showed more foci than were noted grossly. The smaller foci were in the sub-pleural area, not associated with bronchioles, but appeared to arise from the alveolar lining cells as described by Grady and Stewart (1940).

Fig. 5 illustrates an example of multiple foci in the lungs of a female mouse No. 56, 18 months of age, which received 3 × 100 R and 12 urethane injections.

### DISCUSSION

As mentioned in the introduction, the central interest in the present investigation resides in the use of mice of the specific strain denoted X/Gf which do not produce malignant neoplasms, either spontaneously, or after various doses and modes of treatments with X-irradiation. The latter observation is of particular interest, since ionizing radiation has been considered a potent carcinogenic and leukaemogenic agent where induction of these neoplasms in animals is concerned. The interested reader is referred to excellent recent papers on the subject in which references to previous extensive review papers are included (Kaplan, 1964 ; Upton, 1964). Reports pertaining to the induction of various neoplasms by ionizing radiation are continuously and frequently appearing in the literature. Investigators who had employed different strains of mice in radiation studies obtained a high incidence of leukaemias, thymomas (Kaplan, 1964) and other types of neoplasms (Upton, 1964). On the basis of the great variety of neoplasms induced by irradiation, it has been suggested that there are target organs which are relatively more or less susceptible to the induction of this disease. The observations made by us on the X/Gf mice, therefore, present a unique exception in respect to general or target susceptibility to radiation leukaemogenesis or carcinogenesis as a whole.

The mechanism(s) involved in radiation carcinogenesis are not as yet well understood. A recent hypothesis suggests activation of a virus where induction of leukaemias by irradiation is concerned (Kaplan, 1964 ; Berenblum, 1964). This assumption is based on two facts : (a) the induction of leukaemias with cell-free filtrates prepared from leukaemic tissues of irradiated mice and (b) the presence of virus particles in these tissues seen in the electron microscope (Dalton, 1962). This hypothesis finds support in most recent experiments with germ-free mice (Mirand and Grace, 1963 ; DeHarven, 1964 ; Pollard and Matsuzawa, 1964). These experiments showed that leukaemias of lymphocytic type were induced by X-irradiation in germ-free mice and virus-like particles were detected in thymus cells in germ-free mice by the aid of electron microscopy (DeHarven, 1964). Furthermore, the incidence of radiation-induced leukaemias in the germ-free mice was higher than in irradiated conventional mice (Walburg, Upton, Tyndall, Harris and Cosgrove, 1965). Thus, it is assumed that ionizing radiation may activate or potentiate the action of the virus present in the host cells. Kaplan (1964) suggests that radiation may affect the cell harbouring the virus and thereby facilitate its release. Berenblum (1964) postulates that radiation may enhance the maturation of the virus particle. In connection with the aforementioned opinions, the question arises whether the failure of ionizing radiation to induce leukaemias or other types of neoplasms may be explained by an absence of a virus in our X/Gf strain of mice. One pcint in favour of such an assumption is that no virus particles could be detected either in the mammary glands of 4 lactating females or in the thymuses of 3 females and 2 males of the X/Gf mice in the electron microscope. Electron microscopic studies in addition to those mentioned above and of the neoplasms induced by urethane in the X/Gf mice are under way.

The action of urethane presents a different problem. Urethane has proved to be a very potent carcinogen in experimental animals. The term " multipotential " coined by Tannenbaum and Silverstone (1958) on the basis of the results obtained with urethane is justified and finds support in the results herein reported. It can be seen in Tables I to IV that neoplastic lesions were induced in the X/Gf mice of both sexes at various ages. These range from mammary fibroadenomas, mammary carcinomas, lymphomas, leukaemias, undifferentiated sarcoma, and haemangioendotheliomas. It is of significance to note that each type of the above-mentioned neoplastic lesions occurred only in a few instances and then singly in the animals treated either with urethane alone or in combination with

X-irradiation (Tables I to IV). The lung adenomas, however, constitute an exception. These were noted in the majority of the animals of both sexes treated with urethane alone or in combination with X-irradiation. They appeared also in those mice which had developed another type of neoplastic lesion, such as a mammary tumour, lymphoma, or a haemangioma. The incidence of lung adenomas varied from experiment to experiment, depending upon the X-ray and urethane doses as well as the age of the animal. It seems that the variation in incidence of lung adenomas is due mainly to the length of survival of the treated X/Gf mice. It should be recalled from the introduction, that lung adenomas do occur among control, untreated X/Gf mice of both sexes in about 2 to 3% when they are over one year of age. Urethane may act as a potentiating agent in the induction of lung adenomas in X/Gf mice. Such an explanation cannot be offered for the incidence of leukaemias, mammary tumours, or liver haemangiomas, since none of these neoplastic lesions has been noted among control, non-treated mice. The induction of malignant lesions in the X/Gf mice may, therefore, be attributed to the action of urethane alone, particularly since X-irradiation exerted no significant potentiating effect on their incidence. In the case of X/Gf mice, urethane acted as an initiator or as an inducer of these neoplasms in several target organs. The results in Tables I to IV show that the effects of doses of 300 to 400 mg. of urethane were similar to those receiving doses of 40 to 50 mg. per mouse, insofar as incidence of these neoplasms is concerned. The threshold or minimal dose of urethane which would induce neoplasms in the X/Gf mice remains to be determined.

The potentiating effect of X-irradiation on induction of leukaemias by urethane in mice of other strains (Kirschbaum and Kawamoto, 1957 ; Kawamoto, Kirschbaum and Taylor, 1958) failed to appear in the X/Gf mice. The mice used in those experiments usually develop leukaemia spontaneously, though in a low percentage ; it was justified, therefore, to infer that X-irradiation potentiated the occurrence of neoplasms. It was discovered recently that the mice which have been used in these experiments harbour a virus. Thus, the potentiating effect of X-rays in the induction of leukaemia in these mice might be explained, as already mentioned, by the activation of the virus particles by irradiation. Since no leukaemia was induced in the X/Gf mice by X-irradiation alone and no virus particles were detected so far in these mice, it may be inferred that X-irradiation *per se* is not a leukaemogenic agent, at least as far as this strain of mice is concerned. The failure to augment the incidence of spontaneous luekaemias in susceptible AK mice by X-irradiation is another example of lending support to our thesis (Gross, Roswit, Mada, Dreyfuss and Moore, 1959).

The leukaemogenic and carcinogenic action of urethane alone presents another problem. To the knowledge of the writers, the experiments with urethane alone were carried out heretofore on tumour-susceptible mice. For example, Tannenbaum and Silverstone (1958) in their experiments used mice which are inherently susceptible to mammary tumours and these mice are known to harbour the mammary tumour agent or virus. Heston, Vlahakis and Deringer (1960), Deringer (1965), Trainin (1963) and Liebelt, Liebelt and Lane (1964) employed mice which are inherently susceptible to liver cysts or haemangioendotheliomas. Whether these neoplasms carry a virus is not known to the writers. The increased incidence of neoplasms in the aforementioned experiments may, therefore, be due to an enhancing effect of urethane. Conversely, the occurrence of mammary tumours and leukaemias, as well as vascular tumours in the X/Gf mice, though in a very low percentage, may be attributed to the action of urethane as an initiator. Further, as previously mentioned, no virus particles could so far be detected in the mammary glands or thymuses of the X/Gf mice. On the other hand, virus particles were seen in mammary glands of 4-month-old females of a DBA/212 strain of mice also being bred in this laboratory (Gelber, 1963) in which the incidence of mammary tumours is about 90% in breeding females when they reach about one year of age (Goldfeder, Gelber and Moore, 1960).

The mechanism(s) of urethane action is not yet known with certainty. The fact that urethane exerts its effect on a variety of mammalian tissues, regardless of the route of its administration into the animals, i.e., either through drinking water, intraperitoneal injection, by skin painting (Tannenbaun and Silverstone, 1958), or by implating tissues of mice which had been previously treated with urethane (Malmgren and Saxen, 1953), indicates that this chemical diffuses throughout the treated organism, and a minimal amount may be sufficient for its carcinogenic action, since it has been shown that urethane catabolizes rapidly (Mitchell, Hutchinson, Skipper, and Bryan, 1949). Its primary action is supposed to reside in changing the permeability of the cellular membranes (Anselmino and Hoenig, 1930 ; Cornman, 1954) or to act on the genetic material (Dustin, 1963 ; Haddow and Sexton, 1946).

The question why only a few X/Gf mice responded to the carcinogenic action of urethane to produce mammary tumours, lymphomas and vascular tumours remains to be elucidated. The genetic characteristics of these mice are presently under study and this may shed some light on this important problem. Conversely, the high percentage of lung adenomas in the urethane-treated X/Gf mice indicates a local tissue endogenous susceptibility to this disease. In this case, urethane may have acted as a promotor, since lung adenomas, although singly and in only 2 to 3% incidence, were noted in untreated, control X/Gf mice over one year of age.

In discussing local endogenous susceptibility to a specific carcinogenic agent, one should not overlook the possible existence of endogenous resistance to a specific carcinogen. In this connection, reference is made to the failure of urethane to induce pulmonary tumours in white-footed field mice in which pulmonary adenomas do not arise spontaneously (Gross, Gluckman, Kershaw and Posselt, 1953). What the endogenous susceptibility or, conversely, resistance is and how the carcinogen acts upon it remain to be elucidated.

### SUMMARY

This is the first report of a broad investigation on a specific strain of mice, X/Gf, which neither produced malignant neoplasms spontaneously nor after exposure to various doses of X-irradiation, since inbreeding began in 1953. Studies on the combined effects of chemical carcinogens plus X-irradiation were undertaken.

X/Gf mice of various ages were subjected to different doses of urethane plus X-irradiation and to urethane treatments alone. Over 600 mice of similar ages were used as non-treated controls.

Lymphomas appeared in 3·8% of those which were treated with urethane alone and 4·1% treated in combination with X-irradiation. Mammary tumours appeared singly in 4·4% of female mice 6 to 8 months after treatment with urethane alone and 3·2% in combination with X-irradiation. Vascular tumours appeared in 3·8% of mice which had received 12 to 17 injections of urethane alone and in 4·3% treated in combination with X-irradiation (300 to 400 R). Lung adenomas appeared in mice of both sexes either with urethane alone or in combination with X-rays. Significantly higher incidence of lung adenomas occurred among mice which had received 200 R and 4 injections or 4 urethane injections alone than among mice which received 300 or 400 R and 12 to 17 urethane injections.

Among several hundred non-treated control mice, no lymphomas or mammary tumours appeared. Lung adenomas, however, were found in approximately 3% of control non-treated mice about 2 years of age.

Tests on blood pooled from 20 males and 20 females for polyoma virus gave negative results. Electron microscopic examination made so far of mammary glands from 4 breeding females and thymuses of 3 young females and 2 males failed to detect virus-like particles. X-irradiation alone failed to induce malignant neoplasms and also failed to act as an enhancing agent in the induction of leukaemias, mammary tumours, or liver haemangiomas by urethane. Based on this observation, it is inferred that X-irradiation *per se* had no effect as an oncogenic agent on the X/Gf mice. Urethane may be assessed as the sole initiator of the neoplasms which occurred in the X/Gf mice.

The relationship of viral, chemical and radiation carcinogenesis is discussed. Supplementary experiments are in progress.

Grateful appreciation is due to Dr. Robert A. Manaker of the National Cancer Institute, National Institutes of Health, U.S.P.H.S., in whose laboratory the tests for polyoma virus on the blood of X/Gf mice were performed.

The senior author wishes to express her sincere appreciation to the Physics Department of the Department of Hospitals for supplying the physical factors, to Teresa Eilender and to Joel Tricarico for laboratory assistance and to Mrs. June Marks for secretarial help.

This work was supported by Grant U–1354 from the Health Research Council of the City of New York.

### REFERENCES

Anselmino, K. J. and Hoenig, E.—(1930) *Pflügers Arch. ges. Physiol.*, **225**, 56.
Berenblum, I.—(1964) *Acta Un. int. Cancr.*, **20**, 893.
Berenblum, I. and Trainin, N.—(1960) *Science, N.Y.*, **132**, 40.
Cornman, I.—(1954) *Int. Rev. Cytol.*, **3**, 113.
Dalton, A. J.—(1962) *Fedn Proc. Fedn Am. Socs exp. Biol.*, **21**, 936.
DeHarven, E.—(1964) *J. exp. Med.*, **120**, 857.
Deringer, M. K.—(1965) *J. natn. Cancer Inst.*, **34**, 841.
Dustin, P.—(1963) *Pharmac. Rev.*, **15**, 449.
Foley, W. A. and Cole, L. J.—(1964) *Cancer Res.*, **24**, 1910.—(1966) *Radiat. Res.*, **27**, 87.
Gelber, D. F.—(1963) *J. natn. Cancer Inst.*, **30**, 477.
Goldfeder, A.—(1962) *Radiat. Res.*, **16**, 61.—(1965) *Inbred Strains Mice*, **4**, 91.
Goldfeder, A. and Clarke, G. E.—(1956) *Fedn Proc. Fedn Am. Socs exp. Biol.*, **15**, 244.—(1957) *Radiat. Res.*, **6**, 318.—(1957) *Radiat. Res.*, **7**, 51.
Goldfeder, A., Gelber, D. F. and Moore, D. H.—(1960) *J. natn. Cancer Inst.*, **25**, 827.

GRADY, H. G. AND STEWART, H. L.—(1940) *Am. J. Path.*, **16**, 417.
GROSS, L., GLUCKMAN, E. C., KERSHAW, B. B. AND POSSELT, A. E.—(1953) *Cancer, N.Y.*, **6**, 1241.
GROSS, L., ROSWIT, B., MADA, E. R., DRAYFUSS, Y. AND MOORE, L. A.—(1959) *Cancer* Res., **19**, 316.
HADDOW, A. AND SEXTON, W. A.—(1946) *Nature, Lond.*, **157**, 500.
HESTON, W. E., VLAHAKIS, G. AND DERINGER, M. K.—(1960) *J. natn. Cancer Inst.*, **24**, 425.
KAPLAN, H. S.—(1964) *Natn. Cancer Inst. Monogr.*, **14**, 207.
KAWAMOTO, S., KIRSCHBAUM, I. A. AND TAYLOR, G.—(1958) *Cancer Res.*, **18**, 725.
KIRSCHBAUM, A. AND KAWAMOTO, S.—(1957) *Proc. Am. Ass. Cancer Res.*, **2**, 222.
LIEBELT, R. A., LIEBELT, A. G. AND LANE, M.—(1964) *Cancer Res.*, **24**, 1869.
LIVINGOOD, L. E.—(1896) *Johns Hopkins Hosp. Bull.*, **7**, 177.
MALMGREN, R. A. AND SAXEN, E. A.—(1953) *J. natn. Cancer Inst.*, **14**, 411.
MIRAND, E. AND GRACE, J. T.—(1963) *Nature, Lond.*, **200**, 92.
MIRICK, G. S., MCLEAN, J., SMITH, C., LEFTWICH JR., I. AND LEFTWICH, W. M.—(1952) *J. exp. Med.*, **95**, 147.
MITCHELL, J. H., HUTCHINSON, O. S., SKIPPER, H. E. AND BRYAN, C. E.—(1949) *J. biol. Chem.*, **180**, 675.
NETTLESHIP, A., HENSHAW, P. S. AND MEYER, H. L.—(1943) *J. natn. Cancer Inst.*, **4**, 309.
POLLARD, M. AND MATSUZAWA, T.—(1964) *Proc. Soc. exp. Biol. Med.*, **116**, 967.
ROGERS, S.—(1951) *J. exp. Med.*, **93**, 427.
SHIMKIN, M. B.—(1955) *Adv. Cancer Res.*, **5**, 223.
STEWART, H. L.—(1959) ' Pulmonary Tumors in Mice. Physiopathology of Cancer, ' Edited by F. Homburger and W. Fischman, 2nd Edition, New York (Helber and Harper) p. 18.
TANNENBAUM, A. AND SILVERSTONE, H.—(1958) *Cancer Res.*, **18**, 1225.
TRAININ, N.—(1963) *J. natn. Cancer Inst.*, **31**, 1489.
UPTON, A. C.—(1964) *Natn. Cancer Inst. Monogr.*, **14**, 221.
UPTON, A. C., KIMBALL, A. W., FURTH, J., CHRISTENBERRY, K. W. AND BENEDICT, W. H.—(1960) *Cancer Res.*, **20**, 1.
WALBURG, H. E., UPTON, A. C., TYNDALL, R. L., HARRIS, W. W. AND COSGROVE, G. E. —(1965) *Proc. Soc. exp. Biol. Med.*, **188**, 11.